# Aerobic exercise is associated with divergent regulation of macrophage migration inhibitory factor and enhanced remyelination in experimental autoimmune neuritis

**DOI:** 10.1042/BSR20260227

**Published:** 2026-05-29

**Authors:** Yantong Liu, Xiaohan Shi, Ying Wang, Yunhao Deng, Haoyue Chen, Hao Zhang, Yuwen Hao, Ling Zheng, Xiaoyu Wang, Zuncheng Zheng, Kai Wang, Xiaojing Yuan

**Affiliations:** 1 Department of Rehabilitation, Qingdao University affiliated Taian City Central Hospital, Taian 271000, Shandong, China; 2 Binzhou Medical University, Binzhou 256603, Shandong, China; 3 Beichen Hospital of Nankai University, Tianjin 300499, China; 4 Department of Hepatology, Qilu Hospital of Shandong University, Jinan 250012, Shandong, China

**Keywords:** Aerobic exercise, Differential expression, Experimental autoimmune neuritis (EAN), Macrophage migration inhibitory factor (MIF), Myelination, Neuroinflammation

## Abstract

The present study investigated the association between aerobic exercise preconditioning and macrophage migration inhibitory factor (MIF) expression, as well as myelination in the sciatic nerve in experimental autoimmune neuritis (EAN) rats. Female Lewis rats underwent 4-week swimming protocols including high-intensity daily, moderate-intensity daily, and moderate-intensity alternate-day (MOD-AltDay) regimens prior to EAN induction, with control groups of EAN without exercise and Sham operation. Assessment of disease severity, nerve conduction velocity, and sciatic nerve pathology revealed that the moderate-intensity alternate-day exercise regimen significantly delayed disease onset, lowered peak clinical scores, and improved neurological function. Molecular analyses demonstrated that this protective effect was mediated through divergent regulation of MIF: systemic MIF was substantially suppressed (27205.94 ± 4291.76 pg/ml versus EAN 71075.61 ± 10166.41 pg/ml; *P* <0.001) with concomitant reduction in macrophage infiltration, while local MIF expression within the sciatic nerve was significantly elevated (*P* <0.01), correlating with enhanced remyelination as evidenced by increased myelin sheath area (LFB: 67.42 ± 3.26% versus EAN 40.64 ± 9.63%, *P* <0.01) and elevated myelin basic protein expression (0.92 ± 0.14 AU versus EAN 0.59 ± 0.02 AU, *P* <0.05). Crucially, both high-intensity daily and moderate-intensity daily exercise protocols failed to confer comparable benefits. These findings indicate that the protective effect of MOD-AltDay exercise preconditioning on EAN is associated with tissue-specific regulation of MIF, and this change correlates with reduced systemic inflammation and enhanced local remyelination. Pharmacological/genetic studies are needed to confirm mechanisms and evaluate this exercise regimen as a non-pharmacological intervention for autoimmune neuropathy.

## Introduction

Acute inflammatory demyelinating polyneuropathy, clinically referred to Guillain–Barré syndrome (GBS), is an autoimmune disorder of the peripheral nervous system characterized by inflammatory cell infiltration, peripheral nerve demyelination, and axonal injury [1]. Experimental autoimmune neuritis (EAN), a well-established animal model of GBS, faithfully recapitulates the hallmark features of the human condition, including pathological mechanisms, clinical manifestations, and electrophysiological deficits [1]. This model provides a critical platform for investigating disease pathogenesis and potential interventions. Given the acute onset and the risk of irreversible neurological damage associated with GBS and EAN, the development of effective early preventive strategies to attenuate disease severity is of paramount clinical significance.

Aerobic exercise represents an economical, safe, and widely accessible intervention. Beyond enhancing motor function and attenuating muscle atrophy, exercise exerts immunomodulatory and neuroprotective effects, including regulation of inflammatory cytokine release, modulation of immune cell activity, and promotion of nerve repair via enhanced neurotrophic factor secretion [2]. Multiple clinical studies have confirmed that exercise therapy is effective in rehabilitating patients with GBS. Khan et al. found that moderate aerobic exercise can restore motor function of GBS patients, shorten the course of the disease, and reduce fatigue [3]. Garssen et al. conducted 12-week bicycle training for 16 patients with relatively stable neurological function of GBS and found that fatigue levels decreased by 20% and muscle strength significantly improved [4]. Connors et al. reported a case of a COVID-related GBS patient who began progressive rehabilitation training at an early stage of the disease, resulting in a significant recovery of motor function [5]. Other studies also support this conclusion [6,7]. Furthermore, aerobic exercise preconditioning regulates the release of inflammatory factors prior to disease onset, modulates the activity of immune cells, increases the production of neurotrophic factors, and reduces oxidative stress responses, thereby reducing the risk and severity of disease onset. Crucially, the therapeutic efficacy of exercise is highly dependent on both intensity and frequency, underscoring the need for optimized exercise prescriptions. While existing research predominantly focuses on exercise-mediated nerve repair after disease onset—where acute-phase damage may already be irreversible—exercise preconditioning offers a promising prophylactic strategy for GBS-susceptible individuals or those in the infectious prodromal stage. Studies have shown that pre-intervention with aerobic exercise can inhibit the progression of autoimmune demyelinating diseases in the central nervous system [8]. However, systematic studies investigating exercise preconditioning for GBS/EAN prevention are lacking, and the optimal parameters (intensity and frequency) and underlying molecular mechanisms remain poorly defined.

Aerobic exercise not only facilitates the recovery of sensation and motor functions, alleviates pain symptoms caused by injuries, and prevents muscle atrophy but also regulates the release of inflammatory factors, modulates the activity of immune cells, increases the production and release of neurotrophic factors, and promotes the regeneration of damaged nerves. Studies have confirmed that aerobic exercise can regulate the expression of various factors such as IL-10, glial fibrillary acidic protein (GFAP), Iba1, and TNF-α, thereby playing an important role in the rehabilitation process of various diseases [2,9,10]. Macrophage migration inhibitory factor (MIF), a pleiotropic pro-inflammatory cytokine, is widely expressed in immune cells, neurons, and endothelial cells [11]. It plays complex, context-dependent roles in inflammatory responses and tissue repair through activation of multiple signaling pathways. Within the EAN model, MIF exhibits dual functionality: it exacerbates nerve injury and demyelination by recruiting inflammatory cells, yet it also contributes to nerve regeneration processes [12]. This dichotomy highlights MIF’s intricate involvement in peripheral nerve autoimmunity and positions it as a potential key mediator in the interplay between exercise preconditioning and neuroinflammation. Plasma MIF levels are elevated in patients with GBS and correlate positively with disease severity. Moreover, monoclonal antibodies against macrophage MIF or the MIF chemical inhibitor ISO1 can all ameliorate the severity of the disease in EAN mice [13]. The present study suggests that MIF may be a potential target for the treatment of GBS. However, research on MIF in GBS/EAN is still relatively limited. Existing studies mostly focus on the MIF levels in serum or cerebrospinal fluid, which reflect the systemic immune status. However, the local response of GBS/EAN in the peripheral nerves is the core pathological change of the disease, mainly manifested as focal infiltration of inflammatory cells, damage, and regeneration of Schwann cells, among other features. The study of MIF in local nerve tissues is relatively rare. Moreover, the specific role of MIF in mediating the protective effects of exercise preconditioning in EAN has not been systematically explored.

Therefore, the present study aimed to employ the EAN rat model to systematically evaluate the impact of aerobic exercise preconditioning, applied at varying intensities (moderate and high) and frequencies (daily and alternate-day), on neurological outcomes. We assessed functional recovery (clinical scores and nerve conduction velocities), structural integrity (demyelination/remyelination via histopathology), and molecular mechanisms (specifically examining MIF regulation and associated inflammatory factors). This integrated approach, spanning behavioral, pathological, and molecular analyses, seeks to elucidate the potential mechanisms through which optimized exercise preconditioning may prevent or attenuate EAN/GBS, thereby informing the development of targeted exercise-based prophylactic strategies.

## Materials and methods

### Animal groups and exercise preconditioning protocol

A total of 45 female Lewis rats aged 3–4 weeks (with body weights ranging from 70 to 90 g) were obtained from Beijing Vital River Laboratory Animal Technology Co., Ltd. Following a 4-day acclimatization period, the rats were randomly assigned to five experimental groups (*n =* 9 per group) via computer-generated randomization sequence as follows:

EAN control: Received immunization with bovine peripheral nerve myelin P0180-199 peptide (0.67 μg/g emulsified in Complete Freund’s Adjuvant [CFA]; Sigma–Aldrich, Shanghai, China) to induce EAN.

Sham control: Immunized with an equivalent volume of sterile normal saline (0.9% NaCl), serving as the placebo-immunized group.

High-intensity daily exercise (HI-Daily): Underwent 4 weeks of high-intensity daily swimming exercise prior to EAN induction.

Moderate-intensity daily exercise (MOD-Daily): Underwent 4 weeks of moderate-intensity daily swimming exercise prior to EAN induction.

Moderate-intensity alternate-day exercise (MOD-AltDay): Underwent 4 weeks of moderate-intensity swimming exercise every other day prior to EAN induction.

### Exercise preconditioning protocols

Swimming exercise was conducted in temperature-controlled tanks (32 ± 2°C), monitored every 15 min using calibrated thermometers, and maintained by periodic addition of warm water. Water depth exceeded the animals’ body length, ensuring hind limbs could not contact the pool bottom or allow for escape. To maintain continuous activity, rats were gently guided using a rod if they remained immobile for more than 10 s. Post-exercise, animals were immediately towel-dried and placed under a warm air stream until fully dry. Pools were sanitized between sessions.

Three distinct preconditioning regimens were implemented based on established models [14], each spanning 4 weeks (6 days/week unless specified):

#### Moderate-intensity daily exercise

Week 1 (adaptation): Daily swimming. Duration progressively increased: Day 1–2: 15 min; Day 3: 60 min; and Days 4–6: maintained at 60 min/day. No tail loading. Weeks 2–4 (training): 60 min/day of unloaded swimming.

#### High-intensity daily exercise

Week 1 (adaptation): Daily swimming. Duration progressively increased: Day 1–2: 15 min; Day 3: 60 min (unloaded); and Days 4–6: progressive tail loading (1% to 2% to 4% body weight) during 60 min sessions. Weeks 2–4 (training): 60 min/day swimming with tail load equivalent to 4% body weight. Loads were adjusted weekly based on recorded body mass.

#### Moderate-intensity alternate-day exercise

Week 1 (adaptation): Sessions every other day (4 sessions total). Duration progressively increased: Session 1: 15 min; Session 2: 30 min; Session 3: 45 min; and Session 4: 60 min. No tail loading. Weeks 2–4 (training): 60 min sessions of unloaded swimming every other day (3–4 sessions/week).

EAN and Sham control groups remained sedentary throughout the preconditioning period.

### EAN model induction

Following post-exercise preconditioning, EAN was induced in non-sham groups. The bovine P0180-199 peptide (5 mg; Sigma–Aldrich) was dissolved in 2 ml sterile saline (2.5 mg/ml), aliquoted, and stored at −80°C. CFA was prepared by homogenizing *Mycobacterium tuberculosis* H37Ra (20 mg/ml) in incomplete Freund’s adjuvant. The antigen solution was emulsified with an equal volume of CFA via dual-syringe until water-in-oil stability was confirmed. Under isoflurane inhalation anesthesia (1.5%), rats received bilateral hind-paw injections of 0.67 μl/g emulsion (equally split) [15]. Sham controls received sterile normal saline (0.9% NaCl) identically.

### Clinical assessment

Six rats in each group were monitored daily for 45 days post-immunization. Body weight was recorded, and neurological function was assessed using a validated 5-point scale adapted from Moalem-Taylor et al. [16], where scores were defined as follows: 0 indicated normal gait; 1 indicated reduced tail tonicity or limpness; 2 indicated mild hindlimb weakness; 3 indicated moderate hindlimb paralysis; and 4 indicated severe hindlimb paralysis. Intermediate scores (±0.5) were assigned for cases presenting with intermediate clinical signs. Assessments were performed by two independent blinded investigators. The mean score from the two evaluators was used for analysis.

### Exercise tolerance assessment

On day 5 post-immunization, rats were subjected to a 30-min acclimatization swim. Endurance testing was conducted on day 6 using forced swimming with 9% body weight loading. Exhaustion was defined as failure to resurface within 10 s of nasal submersion.

### Neuroelectrophysiology

At the peak of the disease, 3 rats in each group were selected to measure nerve conduction velocity (NCV). Rats were anesthetized by inhalation of 4% isoflurane for induction and maintained under surgical anesthesia with 1.5% isoflurane. Subsequently, the sciatic nerve was surgically exposed. NCV was recorded using a multichannel electrophysiology system. Bipolar stimulating electrodes were positioned on the sciatic nerve proximally. Two recording electrodes, connected to acupuncture needles, were inserted into the gastrocnemius muscle belly. A ground electrode was secured to the hind paw. A supramaximal square-wave stimulus (1 V intensity and 0.1 ms duration) was delivered. NCV was calculated by dividing the distance between recording electrodes by the conduction latency. Following neuroelectrophysiological assessment, rats were administered a lethal dose of isoflurane and then killed by cervical dislocation.

### Mechanistic investigation cohort

Guided by functional outcomes that identified MOD-AltDay as the effective preconditioning regimen (see the ‘Results’ section), a separate cohort of nine female Lewis rats was randomly allocated to three groups (*n* = 3 per group): Sham Control, EAN Control, and MOD-AltDay Exercise (protocols as described above). This cohort was used for molecular and histological analyses at the peak of disease severity.

### Blood and sciatic nerve sampling

At the peak of the disease (day 18 post-immunization), terminal blood was collected from anesthetized rats via cardiac puncture. Blood samples were allowed to coagulate for 30 min at room temperature, centrifuged (3000×***g***, 15 min, 4°C), and serum aliquots were stored at −80°C. After induction of deep anesthesia, rats were killed by cervical dislocation. Bilateral sciatic nerves were then immediately dissected and processed as follows: for transmission electron microscopy, tissues were fixed in ice-cold 2.5% glutaraldehyde at 4°C; for histology (H&E/LFB/IF), tissues were fixed in 4% paraformaldehyde at 4°C; and for Western blotting, tissues were flash-frozen and stored at −80°C.

### Transmission electron microscopy

Rat sciatic nerves were prepared for transmission electron microscopy by dissection and cutting into small blocks of approximately 1 mm^3^. For prefixation, the samples were immersed in a 2.5% glutaraldehyde fixative solution for over 24 h at 4°C. Subsequently, the samples were rinsed three times with 0.1 M phosphate buffer, 10 min each time. Postfixation involved immersion in a 1% osmium tetroxide fixative solution for 3 h at 4°C, followed by rinsing three times with 0.1 M phosphate buffer, 10 min each time. Samples were dehydrated through a graded acetone series (50%, 70%, 80%, 90%, and 100%), with each step lasting 10 min. Infiltration involved mixing acetone with an epoxy resin embedding solution in a 1:1 ratio and allowing the samples to be immersed overnight at room temperature, followed by a 3-h immersion in pure embedding solution at 37°C. Polymerization was carried out by heating the blocks sequentially at 37°C overnight, 45°C for 12 h, and 60°C for 24 h. Ultrathin sections were then prepared using an ultramicrotome to a thickness of 50 nm. These sections underwent double staining with a 3% uranyl acetate solution for 15 min and lead citrate solution for 10 min. Finally, the sections were mounted on electron microscope grids, observed under a transmission electron microscope, and photographed.

### Paraffin section preparation

Sciatic nerves were fixed in 4% paraformaldehyde at 4°C. Fixed tissues were dehydrated through a graded ethanol series (70%, 80%, 90%, 95%, 100%), cleared in xylene, and embedded in paraffin. Sections (8-μm-thick) were cut using a microtome and dried at 60°C for 4 h.

### Hematoxylin–eosin staining

Hematoxylin–eosin (H&E) staining procedure: Deparaffinize with xylene I and II for 10 min each, followed by three 5-min washes in absolute ethanol. Sections were gradually hydrated through a graded series of 95%, 85%, and 50% ethanol, followed by distilled water, for 5 min each. Wash thrice in 1× PBS for 5 min each. Stain with hematoxylin for 5 min, rinse with water for 2 min, check for overstaining, differentiate with 1% hydrochloric acid in ethanol for 3 s, blue with 0.1% ammonia for 30 s, and follow with a 5-min wash under running tap water. Stain with eosin for 1 min and rinse with distilled water for 3 s. Dehydrate with 85%, 95%, and 100% alcohol. Seal with xylene I and II for 5 min each, followed by neutral gum.

### Immunofluorescence staining

The sciatic nerve was fixed with 4% PFA and then serially incubated in 15%, 20%, and 30% sucrose solutions for dehydration. The tissues were embedded in OCT compound, frozen, sectioned, and mounted on slides. The sections were allowed to equilibrate to room temperature for 30 min. The sections were washed three times with 0.01 mmol/l PBS and then incubated with 5% BSA for 1 h at room temperature. The sections were incubated at 4°C overnight with the following primary antibodies: anti-Iba1 (Abcam, ab5076, 1:200) or anti-MIF (Abcam, ab7207, 1:200). After washing three times with 0.01 mmol/l PBS, the secondary antibody Rhodamine (TRITC)-conjugated donkey anti-goat IgG (H+L) (Proteintech, SA00007-3, 1:100) or Cy3-conjugated goat anti-rabbit IgG (Servicebio, GB21303, 1:300) was added in the dark, and the sections were incubated for 1 h at room temperature. After washing three times again with 0.01 mmol/l PBS, the slides were coverslipped with an anti-fade mounting medium containing DAPI and observed under a microscope.

### MIF enzyme-linked immunosorbent assay

Serum MIF levels in rats were measured by double-antibody sandwich enzyme-linked immunosorbent assay (ELISA) following the manufacturer’s instructions (ElaBoXTMRat MIF ELISA Kit, Solarbio, SEKR-0061). The standard and sample wells were designated, and the standards and serum samples were pipetted into the respective wells. The plate was incubated for 2 h at 25°C with shaking. After incubation, the plate was washed. The supernatant was aspirated, and 100 μl of biotinylated detection antibody was added to each well, followed by incubation for 60 min under constant shaking. The plate was washed again, followed by the addition of 100 μl of enzyme conjugate solution to each well, which was incubated with shaking for 30 min. After washing the plate, 100 μl of chromogenic solution was added to each well and incubated in the dark for the recommended duration. Subsequently, 50 μl of stop solution was added to each well. The optical density (OD) value was measured using a microplate reader. A standard curve was plotted using a four-parameter logistic regression model, and the OD value of each sample was measured to calculate its concentration.

### Luxol fast blue staining

The dewaxing and hydration processes are the same as those of HE staining. The sections were placed in Luxol fast blue staining solution and incubated overnight at 37°C. Subsequently, the sections were immersed sequentially in 95% ethanol for 20 s, distilled water for 5 s, lithium carbonate solution for 5 s, 70% ethanol for 15 s, and distilled water for 5 s. If the myelin sheath was not clearly visualized, the sections were repeatedly immersed in lithium carbonate solution and 70% ethanol until the myelin sheath turned blue-green, with a maximum of three repetitions to ensure staining quality. Gradient dehydration was performed by immersing the sections in 95% ethanol for 1 min, followed by absolute ethanol for 3 min. For mounting, the sections were treated with Xylene I for 5 min, then Xylene II for 5 min, followed by the application of a neutral mounting medium.

### Western blot assay

Sciatic nerves stored at −80°C were removed, minced with tissue scissors, and homogenized. After incubating at 4°C for 30 min, the samples were centrifuged at 12,000 rpm for 20 min to extract proteins. Protein concentrations in the samples were quantified using the BCA Protein Quantification Kit. SDS loading buffer and PMSF were added to the samples, which were then diluted to 2 μg/μl. The samples were boiled in a water bath for 5 min to denature the proteins and subsequently stored at −80°C for later use. The proteins were separated by electrophoresis using a 12% SDS-PAGE gel, then electrotransferred to a PVDF membrane. The PVDF membrane was blocked with 5% skim milk blocking solution for 1 h and washed with 1× TBST. The membrane was incubated overnight at 4°C with primary antibodies (mouse anti-myelin basic protein, Abcam, ab62631, 1:1000). After washing the membranes, the membranes were incubated with the secondary antibody (HRP-conjugated goat anti-mouse IgG (H+L), ZSGB-BIO, ZB-2301, 1:5000) for 1 h at room temperature. Following a final wash, protein bands were visualized using a chemiluminescent substrate, and images were captured. Protein bands were quantified using ImageJ.

### Statistical methods

Data were analyzed using GraphPad Prism software (version 10.5). One-way ANOVA was used to compare multiple groups, assuming a normal distribution. Repeated-measures analysis of variance was utilized to compare groups across different time points, and the Bonferroni test was used for post hoc comparisons. Data were expressed as mean ± SD. The Kruskal–Wallis test was applied to data with a non-normal distribution, which are reported as medians (P25, P75). The significance level was set at *P* <0.05.

## Results

### EAN rat model

From as early as the 2nd or 3rd day after immunization, symptoms of lower limb swelling and tail weakness were observed in the EAN group, HI-Daily group, MOD-Daily group, and MOD-AltDay group. Subsequently, the rats’ behavioral scores, based on a standardized neurological assessment scale, gradually increased, and tail drooping appeared. These observations indicate that the EAN rat model was successfully established.

**Figure 1:**
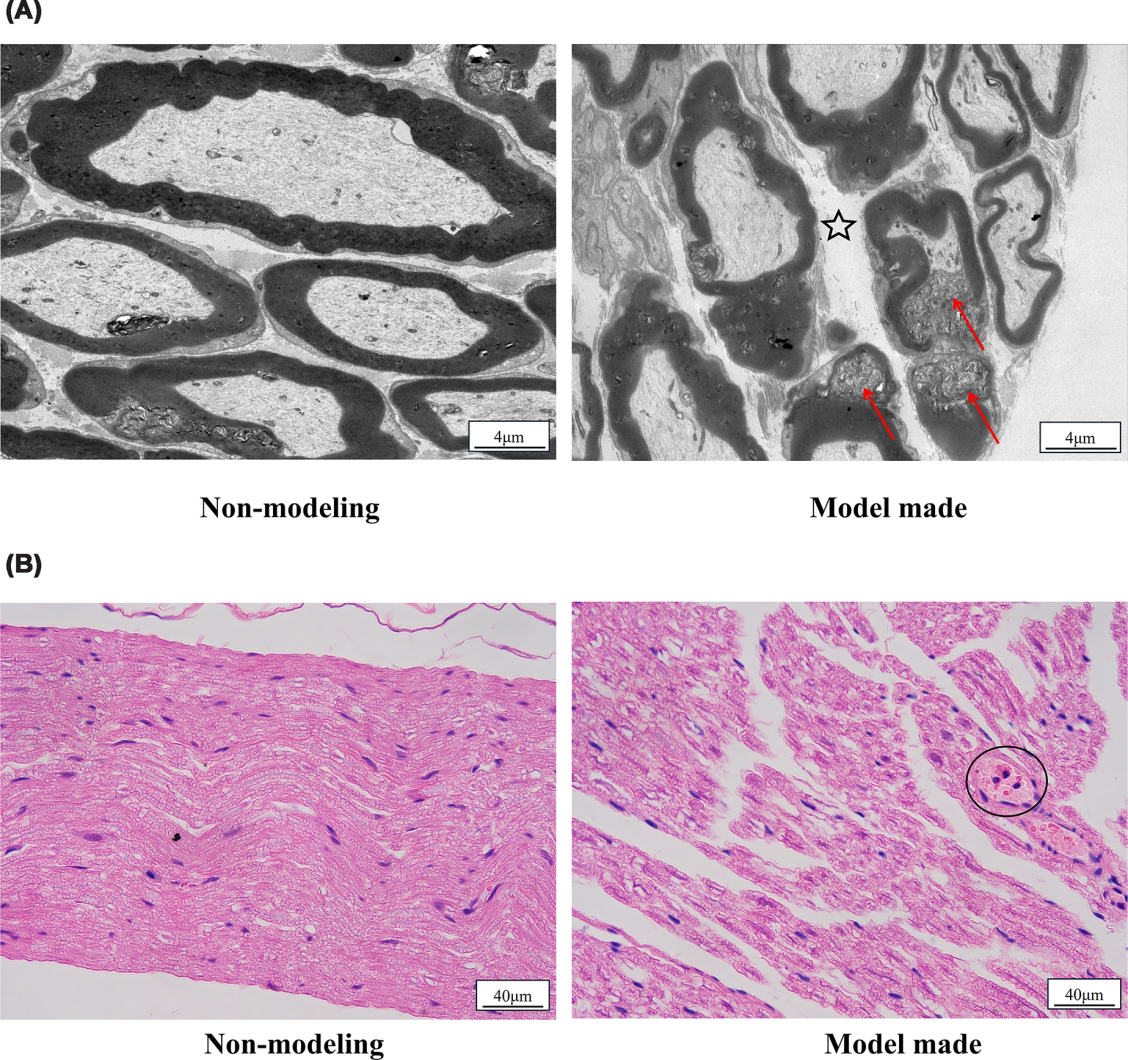
Histopathological comparison of sciatic nerves between non-modeling and EAN-model-made rats. Histopathological comparison of sciatic nerves between non-modeling and EAN-model-made rats. (**A**) Electron micrographs of sciatic nerve cross-sections. Non-modeling rat (left) versus EAN model-made rat at disease peak (right). The EAN model induced severe myelin sheath disruption, including demyelination, localized myelin rupture (arrowhead), and increased extracellular space (asterisk). Scale bar = 4 μm. (**B**) H&E-stained longitudinal sections of sciatic nerves. Non-modeling rat (left) versus EAN model-made rat at disease peak (right). EAN induction resulted in nerve fiber destruction and inflammatory cell infiltration (circle). Scale bar = 40 μm.

At the peak of the disease, ultrastructural examination of the sciatic nerve revealed significant pathological changes, including obvious demyelination and localized myelin sheath ruptures. Additionally, the extracellular space around the myelin sheath was significantly wider than normal. In contrast, the sciatic nerve tissue structure of the non-model rats was preserved, exhibiting clear myelin layers with a uniform and dense arrangement and no significant morphological abnormalities (Figure 1A).

At the disease peak, the sciatic nerve fibers of EAN rats showed varying degrees of damage, characterized by ruptures and swelling of the myelin sheath as well as perivascular infiltration of inflammatory cells. Meanwhile, the sciatic nerve structure of the non-model rats remained intact. The thickness of the myelin sheath was normal, nerve fibers were regularly arranged, the vessel walls were intact, the lumens were regular, and there was no obvious stenosis or dilatation (Figure 1B).

### Determination of effective exercise intensity

#### Clinical score

After EAN induction, the rats began to show symptoms such as swelling of the lower limbs and tail weakness on the 2nd or 3rd day after immunization. Subsequently, the clinical scores of the rats gradually increased, and tail-drooping symptoms appeared. Compared with the EAN group, the onset time of the MOD-AltDay group was significantly delayed (*P* <0.05). However, the onset times for the HI-Daily and MOD-Daily groups did not differ significantly from that of the EAN group (*P* >0.05). At the peak disease severity in the EAN group, the rats exhibited moderate-to-severe hind limb paralysis, and the clinical score reached 4 (3, 4) points. In contrast, the MOD-AltDay group only exhibited tail drooping, hind limb swelling, and mild paralysis, with a clinical score of 2 (2, 2) points, which was significantly lower than that of the EAN group (*P* <0.05). There was no significant difference in clinical scores between the EAN group and the HI-Daily group or the MOD-Daily group, both scoring 4 (4, 4) (*P* >0.05). The peak time of the EAN group was 14.5 (13.75, 14.5) days after immunization. The peak times of the MOD-AltDay group, HI-Daily group, and MOD-Daily group were not statistically different from that of the EAN group (*P* >0.05).

**Figure 2:**
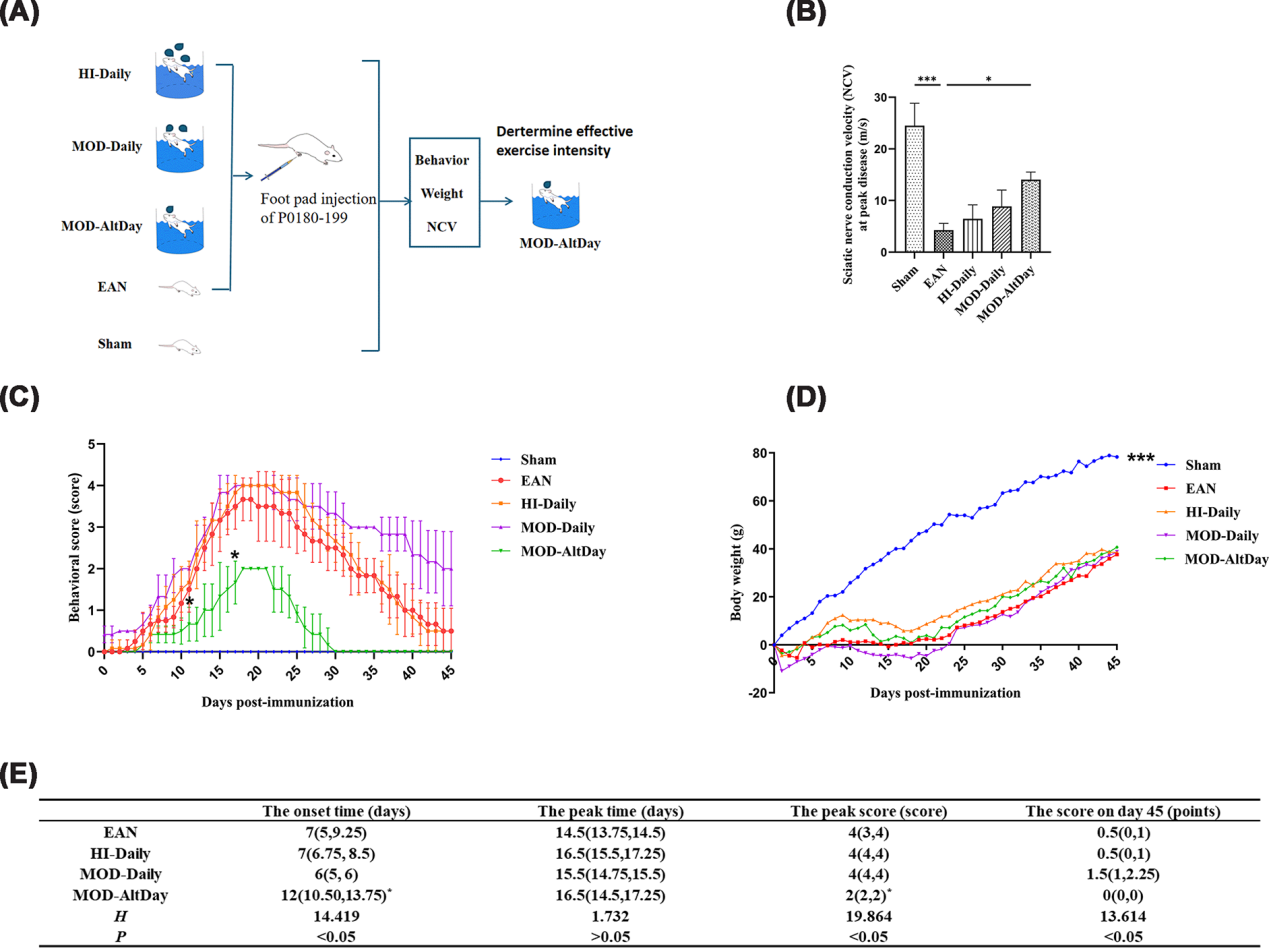
Procedure for determining effective exercise intensity. Effective exercise intensity determination. (**A**) Flow chart. Female Lewis rats underwent 4-week swimming protocols: HI-Daily, MOD-Daily, or MOD-AltDay before EAN induction using P0180–199. Controls included EAN (no exercise) and Sham groups (*n =* 9). MOD-AltDay was determined as effective exercise intensity by disease severity (behavior/weight), and NCVs were assessed at peak disease. (**B**) NCV at peak disease in different groups (*n =* 3). The conduction velocities of other groups were significantly lower than that of the Sham group, which was (24.52 ± 4.3) m/s. The sciatic nerve conduction velocity in the MOD-AltDay group (14.06 ± 1.45 m/s) was significantly higher than that in EAN group (4.31 ± 1.17 m/s) (***P <0.001, EAN group versus Sham group; *P <0.05 MOD-AltDay group versus EAN group). Data are presented as mean ± SD. (C,D) Behavioral and body weight changes post-immunization. Groups: Sham (blue), EAN (red), HI-Daily (orange; high-intensity daily exercise), MOD-Daily (purple; moderate-intensity daily exercise), MOD-AltDay (green; moderate-intensity alternate-day exercise). (**C**) Clinical behavioral scores from day 1 to 45 post-immunization (*n =* 6). The peak time is 16–22 days after induction. The onset time of the MOD-AltDay group was significantly delayed compared with the EAN group (*P <0.05). The peak clinical score of the MOD-AltDay group was significantly lower than that of the EAN group (*P <0.05). (**D**) Body weight progression from day 1 to 45 post-immunization (*n* = 6). The rats in the experimental group showed varying degrees of body weight loss compared with the Sham group (***P <0.001 versus EAN, HI-Daily, MOD-Daily, and MOD-AltDay groups). (**E**) The onset time, peak time, peak clinical behavioral score, and clinical score 45 days post-immunization for each group. Since the data did not follow a normal distribution, the Kruskal–Wallis test was used for analysis. The measurement data were expressed as the median (P25, P75).

On the 29th day, the symptoms of the MOD-AltDay group had completely resolved, with a clinical score of 0 (0, 0), which was significantly lower than that of the MOD-Daily group (*P* <0.05). However, there was no significant difference between the MOD-AltDay group and the EAN group (*P* >0.05). On the 45th day after immune induction, the lower limb function of the EAN group and the HI-Daily group had recovered, with only tail drooping remaining, and the clinical score reached 0.5 (0, 1) (Figure 2C).

In summary, the moderate-intensity alternate-day exercise regimen attenuated the disease course, as evidenced by a significant delay in onset, a reduction in peak clinical scores, and a faster recovery.

#### Changes in body weights

Following immune activation to simulate disease conditions, the rats in the experimental group exhibited significant body weight loss compared with the sham group; this difference in body weight was statistically significant (*P* <0.001). Furthermore, compared with the EAN group, body weights did not differ significantly among the MOD-daily group (daily moderate), the HI-daily group (daily high-intensity), and the MOD-AltDay group (alternate-day moderate) (*P* >0.05) (Figure 2D).

#### Endurance test

On the 6th day after immune induction, the endurance capacity of the HI-Daily, MOD-Daily, and MOD-AltDay groups was higher than that of the EAN group. However, the differences between these groups and the EAN group were not statistically significant (*P* >0.05).

#### Electrophysiological function of the sciatic nerve

During the peak stage of the disease, the sciatic nerve conduction velocities in the HI-Daily group, MOD-Daily group, MOD-AltDay group, and EAN group were (6.46 ± 2.7) m/s, (8.86 ± 3.17) m/s, (14.06 ± 1.45) m/s, and (4.31 ± 1.17) m/s, respectively. These conduction velocities were significantly lower than those of the sham group, which were (24.52 ± 4.3) m/s (*P* <0.05, *P* <0.001). However, there was no significant difference in sciatic nerve conduction velocity between the HI-Daily group and the MOD-Daily group and EAN group (*P* >0.05). In contrast, the sciatic nerve conduction velocity in the MOD-AltDay group was significantly higher than that in the EAN group (*P* <0.05) (Figure 2B). Consequently, the moderate-intensity alternate-day exercise regimen improved neurological function, as assessed by electrophysiological evaluation.

### Aerobic exercise alters inflammatory factor expression and enhances myelination in EAN rats

In the preceding experiments, the effective exercise intensity for EAN was established, and the intervention’s beneficial effects on EAN rats were evaluated based on behavioral scores and electrophysiological results (see the ‘Results’ section). Furthermore, analyses including sciatic nerve LFB staining, immunofluorescence staining, and Western blotting were conducted, and serum MIF levels were quantified. Specific results were as follows:

#### Immunofluorescence for MIF in the sciatic nerve and ELISA for MIF in the serum of rats

**Figure 3:**
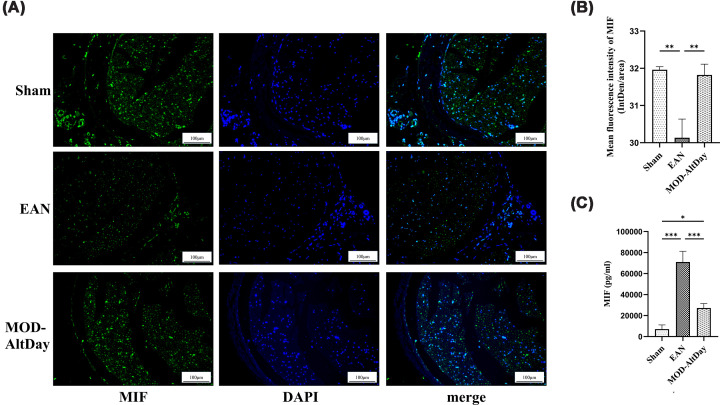
MIF immunofluorescence of sciatic nerve and MIF ELISA results in serum in Lewis rats at peak disease. MIF immunofluorescence of sciatic nerve and MIF ELISA results in serum in Lewis rats at peak disease in MOD-AltDay group and control group (Sham and EAN). (**A**) Representative images showing MIF immunofluorescence of sciatic nerve (scale bar = 100 μm). (**B**) Statistical results of immunofluorescence for MIF of sciatic nerve (*n =* 3). The MOD-AltDay group showed statistically significant increase in the expression of MIF in the sciatic nerve compared with that in the EAN group on day 18 p.i. which is the peak phase of EAN. (**P <0.01 EAN group versus Sham group and MOD-AltDay group.) Data are presented as mean ± SD. Sham group: (31.96 ± 0.083) (IntDen/area), EAN group: (30.13 ± 0.50) (IntDen/area), and MOD-AltDay group: (31.82 ± 0.29) (IntDen/area). (**C**) Statistical results of Elisa for serum MIF (*n =* 3). The MOD-AltDay group showed statistically significant reduction in the concentration of MIF in the serum compared with that in the EAN group on day 18 p.i. (***P <0.001, EAN group versus Sham group and MOD-AltDay group; *P <0.05, EAN group versus Sham group). Data are presented as mean ± SD. Sham group: (7165.29 ± 3955.04) pg/ml, EAN group: (71075.61 ± 10166.41) pg/ml, and MOD-AltDay group: (27205.94 ± 4291.76) pg/ml.

ELISA results showed that 18 days after induction, the serum MIF concentrations in the sham group, EAN group, and MOD-AltDay group were (7165.29 ± 3955.04) pg/ml, (71075.61 ± 10166.41) pg/ml, and (27205.94 ± 4291.76) pg/ml, respectively. Serum MIF concentrations in both the EAN and MOD-AltDay groups were significantly higher than those in the sham group (*P* <0.001 and* P* <0.05, respectively). Furthermore, the serum MIF concentration in the MOD-AltDay group decreased by 38.3% compared with the EAN group (*P* <0.001) (Figure 3C). However, immunofluorescence analysis revealed that MIF expression in the sciatic nerve of the MOD-AltDay group was significantly higher than that of the EAN group (*P* <0.01; Figure 3A,B). These results indicate that MOD-AltDay aerobic exercise exerts a bidirectional regulatory effect on MIF, substantially suppressing its systemic levels while concurrently up-regulating its local expression in the sciatic nerve.

#### Immunofluorescence for Iba-1 in the sciatic nerve

**Figure 4:**
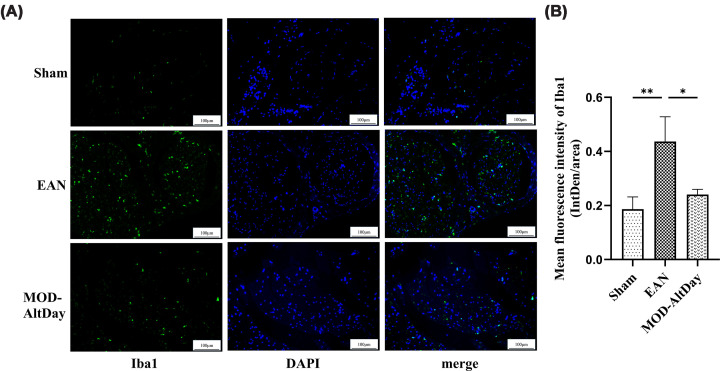
Iba-1 immunofluorescence of sciatic nerve in Lewis rats at peak disease. Iba-1 immunofluorescence of sciatic nerve in Lewis rats at peak disease in the MOD-AltDay group and control group (Sham and EAN). (**A**) Iba-1 immunofluorescence results of the sciatic nerve (scale bar = 100 μm). (**B**) Quantitative analysis of Iba-1 immunofluorescence in the cross-sections of the sciatic nerve in each group (*n =* 3). The MOD-AltDay group showed a statistically significant reduction in the average fluorescence intensity of Iba-1 compared with that in the EAN group on day 18 p.i. (**P <0.01, EAN group versus Sham group; *P <0.05, MOD-AltDay group versus EAN group). Data are presented as mean ± SD. Sham group: (0.19 ± 0.05) (IntDen/area), EAN group: (0.4 ± 0.09) (IntDen/area), and MOD-AltDay group: (0.24 ± 0.02) (IntDen/area).

To explore the changes in inflammatory response in the sciatic nerve of EAN rats after aerobic exercise as a pre-intervention, we detected the expression level of the macrophage marker Iba1 in transverse sections of the sciatic nerve by immunofluorescence on the 18th day after immune challenge in the sham, EAN, and MOD-AltDay groups. The expression of Iba-1 was increased in the EAN group compared with the sham groups (*P* <0.01). Conversely, the MOD-AltDay group exhibited reduced Iba-1 expression levels compared with the EAN group, suggesting that MOD-AltDay aerobic exercise inhibits macrophage activation (*P* <0.05; Figure 4).

#### LFB staining of the sciatic nerve

The sciatic nerve of rats in the sham group had an intact myelin sheath by LFB staining, showing a typical blue myelin sheath with a clear outline and uniform staining. At the peak of the disease (18 days post-immune induction), the EAN group exhibited disrupted myelin sheaths and uneven staining. Some areas of myelin staining appeared markedly lighter blue, suggesting nerve damage and demyelination. In the MOD-AltDay group, some areas appeared lighter blue, the degree of demyelination was less severe, and the size of demyelinated areas was smaller than that in the EAN group.

**Figure 5:**
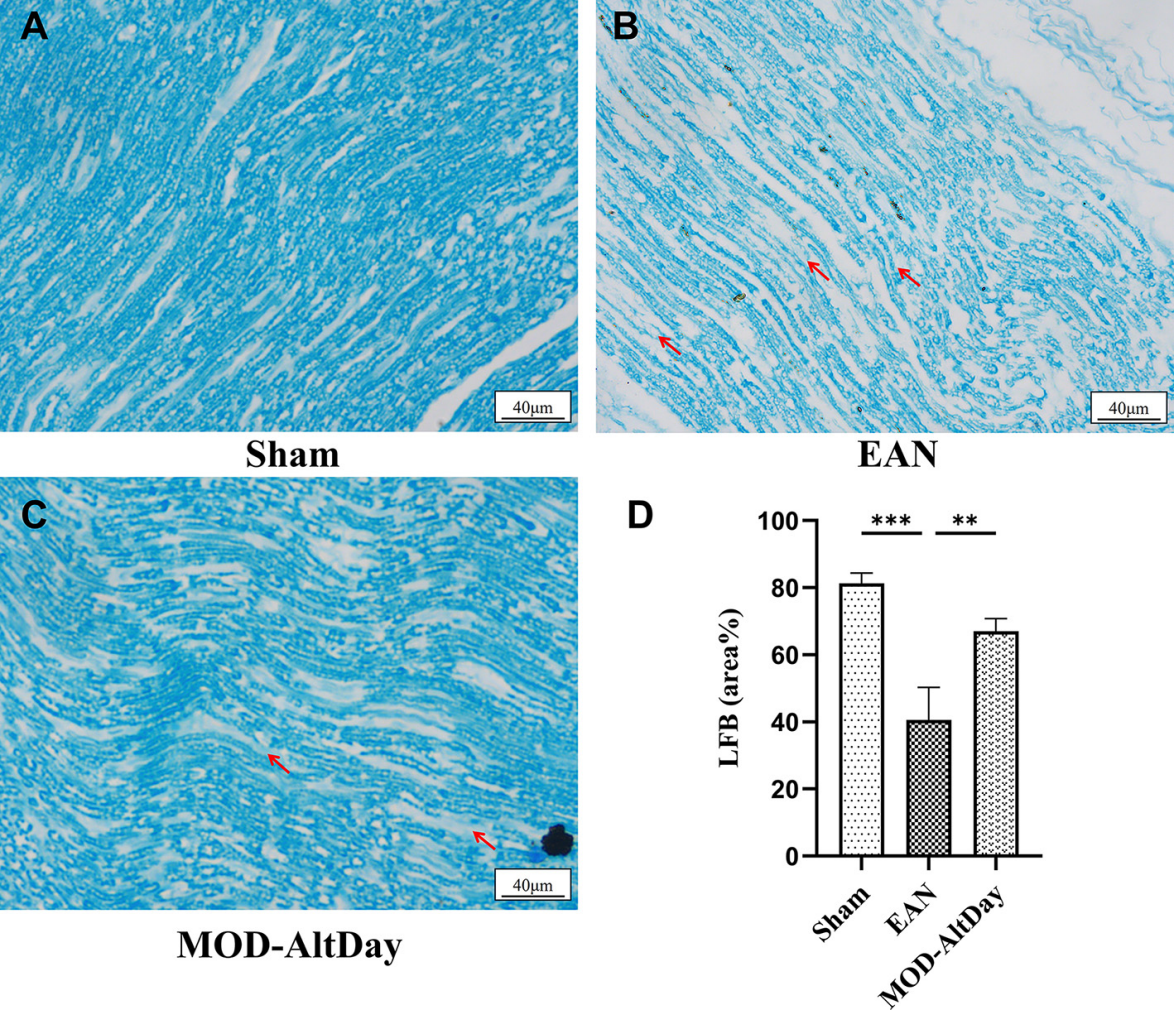
Sciatic nerve LFB staining in Lewis rats at peak disease. Sciatic nerve LFB staining in Lewis rats at peak disease in the MOD-AltDay group and control group (Sham and EAN). (**A**–**C**) LFB staining results showed disrupted myelin sheath and uneven staining (arrowhead) (scale bar = 40 μm). (**D**) LFB staining quantitative analysis (*n =* 3). The MOD-AltDay group showed a statistically significant reduction in sciatic nerve demyelination compared with the EAN group on day 18 p.i. (***P <0.001, EAN group versus Sham group; **P <0.01, MOD-AltDay group versus EAN group). Data are presented as mean ± SD. Sham group: (81.32 ± 3.05)%, EAN group: (40.64 ± 9.63)%, and MOD-AltDay group: (67.42 ± 3.26)%.

The relative areas of myelin staining in the sciatic nerve of the sham, EAN, and MOD-AltDay groups were 81.32 ± 3.05%, 40.64 ± 9.63%, and 67.42 ± 3.26%, respectively. The degree of demyelination in the EAN group was significantly higher than that in the sham group (*P* <0.001). Moreover, compared with the EAN group, the degree of demyelination was significantly reduced in the MOD-AltDay group, indicating the inhibitory effect of MOD-AltDay aerobic exercise on peripheral nerve demyelination (*P* <0.01) (Figure 5).

#### MBP expression in the sciatic nerve by western blot

**Figure 6:**
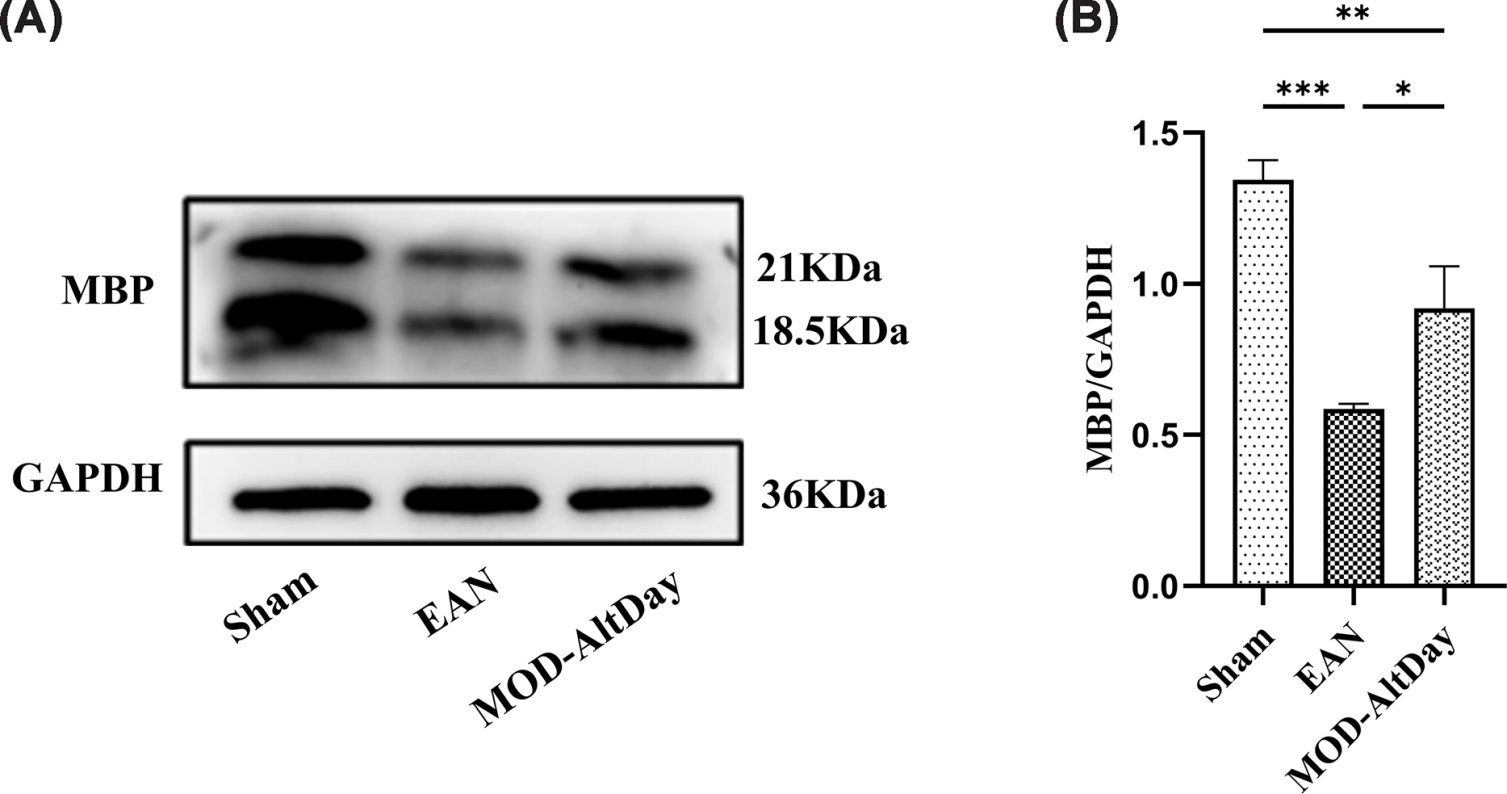
Western blot (WB) analysis of MBP in the sciatic nerve in Lewis rats at peak disease. Western blot (WB) analysis of MBP in the sciatic nerve in Lewis rats at peak disease in MOD-AltDay group and control group (Sham and EAN). (**A**) WB results of MBP in sciatic nerve. (**B**) The relative expression level of MBP in sciatic nerve by WB (*n =* 3). The MOD-AltDay group showed a statistically significant increase in the gray value of the WB band of MBP compared with that in the EAN group on day 18 p.i. (***P <0.001, EAN group versus Sham group; *P <0.05, MOD-AltDay group versus EAN group; and **P <0.01, MOD-AltDay group versus Sham group). Data are presented as mean ± SD. Sham group: (1.34 ± 0.06), EAN group: (0.59 ± 0.02), and MOD-AltDay group: (0.92 ± 0.14).

Western blot analysis performed that 18 days post-immune induction revealed gray-scale values of 1.34 ± 0.06, 0.59 ± 0.02, and 0.92 ± 0.14 for the sham, EAN, and MOD-AltDay groups, respectively. The expression of MBP in the MOD-AltDay group was 1.56-fold higher than that in the EAN group. The difference was statistically significant (*P* <0.05), indicating that MOD-AltDay aerobic exercise inhibited demyelination and promoted myelination and repair (Figure 6).

## Discussion

GBS is an immune-mediated disease of the peripheral nervous system. The typical pathological changes are peripheral nerve demyelination, axonal injury, and inflammatory cell infiltration. The most characteristic clinical manifestation in patients with GBS is motor dysfunction of varying degrees. Globally, GBS is considered the most common cause of acute flaccid paralysis following the decline in poliomyelitis incidence [1]. Although symptoms improve in most patients following systematic treatment, approximately 20% retain varying degrees of dysfunction one year after disease onset, while some fail to regain independent walking ability, significantly impacting their daily living and occupational functioning [17]. Therefore, investigating more effective rehabilitation interventions for GBS is clinically significant for improving patient prognosis and quality of life.

EAN is a classic animal model of GBS, and its main pathological features include macrophage and lymphocyte infiltration and demyelination of peripheral nerves [18]. The EAN model was first established by Waksman and Adams, who injected the sciatic nerve or spinal ganglion of rabbits to induce a disease where inflammation was confined to the peripheral nervous system [19]. Subsequent studies successfully induced the EAN model in many different animal strains by combining adjuvants and various purified antigens [20]. Due to the simplicity of the experimental operation in rats, the similarity of the nervous system structure to that of humans, and the availability of a large amount of verified data, Lewis rats were selected as the experimental animals for model induction in the present study.

Aerobic exercise is widely applied in the rehabilitation of various diseases. Aerobic exercise not only helps restore sensory and motor functions, alleviates pain symptoms caused by injuries, and prevents muscle atrophy but also regulates the release of inflammatory factors, modulates the activity of immune cells, enhances the synthesis and secretion of neurotrophic factors, and promotes the regeneration of damaged nerves. For instance, studies have shown that exercise can specifically regulate the expression of Toll-like receptors in the immune cells of subjects, suppressing excessive immune responses, thereby reducing inflammatory responses and preventing the further deterioration of nerve damage [21]. Studies on patients with multiple sclerosis have also confirmed that aerobic exercise can promote the secretion of anti-inflammatory cytokines (such as IL-10), thereby enhancing the immune regulatory ability of patients and reducing inflammatory responses [22]. In animal model studies, aerobic exercise has a significant effect on the recovery of peripheral nerve injury and central nervous system injury. For instance, Kong et al. found that the expression of tumor necrosis factor (TNF) was inhibited in rats with severe traumatic peripheral nerve injury after treadmill exercise. This treatment improved axonal alignment and promoted nerve regeneration [2]. Zheng et al. demonstrated that roller exercise in mice enhanced the proliferative capacity of oligodendrocyte precursor cells, thereby promoting myelin formation in the motor cortex [23]. Leite et al. demonstrated that swimming exercise can inhibit up-regulation of the microglial cell marker Iba1 and GFAP levels as well as the down-regulation of the neuronal markers NeuN level in the hypothalamus of elderly rats. It can reduce inflammatory responses and also inhibit the proliferation of astrocytes and the activation of microglia in the hypothalamus, thereby exerting neuroprotective effects [10].

Behavioral scores and body weight were monitored daily for 45 days after induction. Our results suggest that moderate exercise every other day can best improve the symptoms of EAN rats and promote disease remission, while excessive exercise intensity and high frequency may exacerbate EAN symptoms. The exhaustion test was conducted during the disease phase in the rats. According to the statistical results, compared with the EAN group, the average endurance of the HI-Daily group, MOD-Daily group, and MOD-AltDay group showed an upward trend, but the difference was not statistically significant (*P* >0.05). Future studies should involve a larger sample size. Slowed nerve conduction velocity indicates nerve demyelination [24]. NCV was measured at the peak of the disease. The results showed that demyelination of the sciatic nerve gradually occurred with the progress of the disease, which slowed down the conduction velocity of the sciatic nerve. Moderate-intensity aerobic exercise every other day could inhibit the demyelination of the sciatic nerve. Further studies should be conducted to measure more sensitive indicators such as F wave and H reflex to comprehensively evaluate the recovery of neurological function.

EAN rat model was successfully established in the present study, which provides a reliable basis for further study on the recovery of GBS and its mechanism. The results of the present study showed that alternate-day moderate-intensity aerobic exercise was better able to reduce symptoms and improve motor function in EAN rats compared with daily high-intensity aerobic exercise and daily moderate-intensity aerobic exercise, thus confirming it as an effective exercise intervention dose. On this basis, sciatic nerve LFB staining, immunofluorescence staining, WB, and serum MIF detection were performed in the sham group, the EAN group, and the MOD-AltDay group to further evaluate the effect of aerobic exercise intervention on nerve repair, myelin reconstruction, and immune system regulation in the EAN group.

MBP is a membrane protein that is widely present in the myelin sheath of the peripheral and central nervous systems [25] and is involved in the formation of myelin. In animals lacking MBP, the diameter of axons and the thickness of myelin sheaths are significantly reduced. Following nerve injury, the MBP content at the injury site decreases, suggesting that the integrity of the myelin sheath is affected [26]. The extent of nerve damage and repair can be better assessed by monitoring changes in MBP expression. The results of MBP WB showed that aerobic exercise could promote the expression of MBP protein, inhibit demyelination, and promote the formation and repair of myelin sheath. The results of LFB staining further indicated that aerobic exercise could promote the activity and proliferation of Schwann cells and inhibit the destruction of myelin sheath.

Macrophages are critical immune cells derived from embryonic progenitor cells or monocytes and exist in various tissues of the body. They play an important role in both innate and adaptive immunity [26]. Macrophages are highly plastic and able to express different phenotypes in response to distinct environmental signals [27,28]. In GBS, Th1 cells can induce the activation of M1-type macrophages, and M1 cells can promote the expression of major histocompatibility complex class II (MHC-II) molecules, adhesion molecules, and inflammatory cytokines. The high expression of MHC-II enables macrophages to specifically present antigens to T cells, leading to the occurrence of inflammatory responses [29]. The adhesion molecules expressed in macrophages play a critical role in the aggregation and activation of T cells [30], thereby promoting the demyelinating process of EAN [31]. During the recovery phase of GBS, M2-type macrophages play a neuroprotective role. Nerve damage can lead to the deposition of collagen VI and apolipoprotein E, thereby activating M2-type macrophages [32,33], controlling inflammation, clearing myelin and axon fragments, and promoting axon and myelin regeneration [33,34]. The results of immunofluorescence showed that EAN-induced inflammatory response could lead to the activation of macrophages, thereby contributing to the process of nerve injury and immune-mediated demyelination. MOD-AltDay aerobic exercise may suppress the inflammatory response, attenuate the excessive macrophage activation and mediator release, and mitigate nerve injury.

Macrophage MIF is a pleiotropic proinflammatory cytokine and immunomodulator, which is widely expressed in immune cells, nerve cells, epithelial cells, smooth muscle cells, and other cells [35]. MIF has a dual role in GBS. MIF can induce the up-regulation of Toll-like receptor 4 (TLR4), regulate the immune response induced by lipopolysaccharide [31], promote the activation and recruitment of immune cells, and participate in the pathogenesis of GBS. MIF can increase the expression of TNF-α, IL-12, IL-6, and other pro-inflammatory cytokines [36], thereby inducing demyelination of peripheral cells. MIF also has chemotactic properties. MIF interacts with the CXCR2 and CXCR4 receptors to trigger these effects [37], promote the migration of monocytes, T cells, and neutrophils, and facilitate the occurrence of an immune response [38]. However, MIF also exhibits neuroprotective effects. MIF can accelerate peripheral nerve regeneration and prevent Schwann cells from apoptosis by inhibiting the accumulation of p53 [39]. thereby promoting the growth, survival, and regeneration of damaged axons. It can also activate ERK/MAPK and PI3K/AKT signaling pathways [40,41]. Thus, it modulates the transcriptional and post-transcriptional regulation of cytokine gene expression, which in turn leads to neural cell proliferation [42]. The activation of Akt not only provides survival signals for cells to prevent apoptosis but is also related to nerve regeneration and the axonal growth of motor neurons [40].

Nicoletti et al. conducted a study involving both patients with GBS and mice with EAN, revealing that plasma MIF levels were elevated in GBS patients and correlated positively with disease severity. Moreover, anti-macrophage MIF monoclonal antibody or MIF chemical inhibitor ISO1 can alleviate disease severity in mice with EAN [13]. The present study suggests that MIF is a promising therapeutic target for GBS. Further research may lead to the development of novel MIF-targeted therapeutic strategies, offering more effective treatment options for patients with GBS.

The results of serum ELISA detection in this experiment showed that during the peak period of the disease, the serum MIF of rats in the EAN group and the MOD-AltDay group increased compared with the sham group. This indicates that EAN can induce the production of MIF, which is consistent with the research results of Nicoletti et al. The serum MIF level of rats in the MOD-AltDay group was lower than that in the EAN group, suggesting that aerobic exercise has a regulatory effect on the inflammatory response of the body. Aerobic exercise may attenuate the systemic inflammatory response, mitigate peripheral nerve damage, and facilitate nerve repair by modulating immune function and suppressing excessive immune activation.

Although serum MIF levels in patients with GBS after aerobic exercise have not been fully elucidated, previous studies have demonstrated that aerobic exercise has a regulatory effect on MIF in other diseases, providing a rationale for the present study. Chan et al. reported that aerobic exercise increased MIF expression levels in the peri-infarct region of patients with ischemic stroke. The authors used the relative FA value (rFA) of DTI in MRI to evaluate the degree of ischemic injury, and the increase in rFA value indicated that the degree of injury was reduced. The results of the present study showed that the expression level of MIF was correlated with rFA value, suggesting that aerobic exercise may inhibit cell apoptosis and promote nerve repair by increasing the expression level of MIF. Moreover, the sham-operated group in the present study also showed an increase in MIF levels after aerobic exercise [43], suggesting that aerobic exercise can increase MIF expression in healthy people.

In contrast with the results of the present study, which showed that appropriate aerobic exercise decreased serum MIF levels in EAN rats, MIF levels increased after aerobic exercise in both ischemic stroke and healthy people. This discrepancy may be attributed to the different regulatory effects of aerobic exercise on the immune system in different models and the different mechanisms of MIF action. In stroke and healthy individuals, aerobic exercise promotes immune cell activity and inflammatory response, leading to increased MIF levels, enhanced nerve repair, and anti-inflammatory effects. The immune system of EAN rats is over-activated in the early stage, leading to myelin damage, neuroinflammation, and immune cell migration. An excessive increase in MIF can aggravate the inflammatory response, leading to nerve damage and loss of function. Moderate aerobic exercise can regulate the level of inflammation in the body, reduce the excessive immune response of EAN, reduce the level of MIF, and thus reduce the attack of immune cells on the nerve. This may help to reduce the inflammatory response and improve recovery after nerve injury.

The immunofluorescence results of this experiment showed that the MIF levels in the sciatic nerves of rats in the EAN group were significantly lower than those in the Sham group and the MOD-AltDay group, presenting an opposite trend to serum MIF levels. Possible reasons are as follows: Research by Nishio et al. indicates that in the sciatic nerves of healthy rats, MIF protein is localized in Schwann cells [44]. Schwann cells are crucial glial cells in the peripheral nervous system, constituting approximately 70% of all peripheral nerve cells [45]. They play vital roles in nerve development, myelin formation, nerve repair, and regeneration. Derived from neural crest cells, Schwann cells exert key functions during early development, postnatal stages, and adulthood. Under physiological conditions, they provide protection and nutritional support by tightly enveloping axons, meeting their energy demands to ensure normal axonal function and neuronal survival [46]. Consequently, MIF expression remains relatively stable in highly mature Schwann cells within the sham group. Under pathological conditions in EAN rats, activated T cells infiltrate peripheral nerves and locally release potent inflammatory signals such as high levels of interferon-γ (IFN-γ) [12]. Stimulated by strong inflammatory signals like IFN-γ, Schwann cells can transform into antigen-presenting cells, up-regulating the expression of molecules such as major histocompatibility complex class II (MHC class II) and intercellular adhesion molecule-1, thereby participating in T cell activation [47]. This immune activation state may reduce Schwann cell synthesis of MIF. Concurrently, Schwann cells may also release large amounts of intracellularly stored MIF. On the one hand, MIF exerts chemotactic effects by interacting with CXCR2 and CXCR4 receptors, promoting the migration of monocytes, T cells, and neutrophils to trigger local inflammatory responses [37]. On the other hand, MIF induces up-regulation of TLR4, modulating lipopolysaccharide-induced immune response [41]; promoting immune cell activation and recruitment; and participating in the pathogenesis of GBS. Consequently, MIF is secreted and consumed in large quantities and continuously, while the rate of synthesis may fail to meet demand. This leads to decreased MIF levels in the sciatic nerve, resulting in weak signals detected by immunofluorescence. Aerobic exercise pre-intervention helps maintain Schwann cell functional stability and inhibit myelin loss by alleviating inflammatory responses in EAN rats. Consequently, the reduction in MIF levels in the sciatic nerves of the exercise group rats was significantly suppressed in this experiment.

In conclusion, moderate-intensity aerobic exercise every other day can significantly improve the motor function of EAN rats, promote the repair of sciatic nerve myelin, and accelerate the recovery of NCV. Our findings suggest an association between the beneficial effects and region-specific MIF regulation patterns: systemic MIF levels were decreased (potentially linked to reduced inflammation), while local MIF in the sciatic nerve was increased. These correlative observations are accompanied by reduced macrophage infiltration, enhanced myelin regeneration, and improved neurological function. However, daily high-intensity and daily moderate-intensity exercise showed no significant protective effects, suggesting a dose-dependent effect of exercise. The present study provides an experimental basis for optimizing the rehabilitation training program of EAN and provides a reference for the prevention strategy of GBS-susceptible and prodromal populations.

### Deficiencis and prospects

Several limitations of the present study should be acknowledged. First, no pharmacological or genetic approaches (e.g., MIF knockout, neutralizing antibodies, or agonists) were employed to directly validate the causal role of MIF in the observed protective effects. Therefore, the association between exercise-induced MIF changes and myelination should be interpreted as correlational rather than causal. Second, the downstream signaling pathways through which MIF might exert its effects—such as CXCR2, CD74, or other chemokine axes—were not investigated, and the potential involvement of other mediators (e.g., IL-6, TNF-α, and oxidative stress markers) remains unexplored. Third, although remyelination was observed, apoptosis of Schwann cells or inflammatory cells was not assessed, limiting the understanding of cell survival dynamics. Fourth, the exercise preconditioning model (intervention initiated prior to disease induction) differs from the clinical scenario of GBS, where patients typically present after symptom onset. Consequently, the specific exercise parameters used in this EAN model cannot be directly translated to acute GBS patients. Fifth, this is a preclinical animal study without human data; the translational relevance of tissue-specific MIF regulation to human GBS remains to be established.

Despite these limitations, the present study provides several directions for future research. Given the correlational nature of our findings, future studies should employ pharmacological (e.g., MIF-specific inhibitors) or genetic (e.g., MIF knockout or overexpression) approaches to determine whether MIF plays a causal role in exercise-mediated neuroprotection. Additionally, investigation of downstream signaling pathways (e.g., CD74/CXCR4, ERK, and NF-κB) and alternative mediators (e.g., other cytokines, chemokines, or oxidative stress markers) will help elucidate the broader regulatory network. Regarding clinical translation, although the preconditioning paradigm does not directly mirror acute GBS management, our observation of tissue-specific MIF changes associated with exercise provides a theoretical basis for designing exercise prevention programs and post-onset exercise interventions. Future research should (i) validate these findings in human samples (e.g., serum and cerebrospinal fluid from GBS patients); (ii) determine the optimal timing (e.g., during the recovery phase rather than the acute phase), intensity, and frequency of exercise that safely promote remyelination; and (iii) develop feasible rehabilitation protocols for GBS patients with varying degrees of motor impairment. Finally, the EAN model remains a valuable platform for mechanistic discovery, but findings should be validated in other animal models (e.g., non-active immunization models) and, ultimately, in well-designed clinical cohort studies before clinical application.

## Data Availability

The datasets generated and/or analyzed during the current study are available from the corresponding author upon reasonable request and are also included within the article and its supplementary files, including the original, uncropped Western blot images in Supplementary File S1.

## Abbreviations

CFA: complete Freund’s adjuvant
ELISA: enzyme-linked immunosorbent assay
EAN: experimental autoimmune neuritis
GFAP: glial fibrillary acidic protein
GBS: Guillain–Barré syndrome
H&E: hematoxylin–eosin
HI-Daily: high-intensity daily exercise
IFN-γ: interferon-γ
MHC-II: major histocompatibility complex class II
MIF: migration inhibitory factor
MOD-AltDay: moderate-intensity alternate-day
MOD-Daily: moderate-intensity daily exercise
NCV: nerve conduction velocity
OD: optical density
rFA: relative FA value
TNF: tumor necrosis factor
TLR4: Toll-like receptor 4
WB: Western blot
